# Supplementary material for: Berberis microphylla G. Forst Intake Reduces the Cardiovascular Disease Plasmatic Markers Associated with a High-Fat Diet in a Mice Model
Source: Antioxidants (Basel). 2023 Jan 28;12(2):304. doi: 10.3390/antiox12020304 (PMC9952125; doi:10.3390/antiox12020304)
Supplement: Supplementary file 1 [file antioxidants-12-00304-s001.zip › Supplementary material 1 rev def.pptx]

## Slide 1
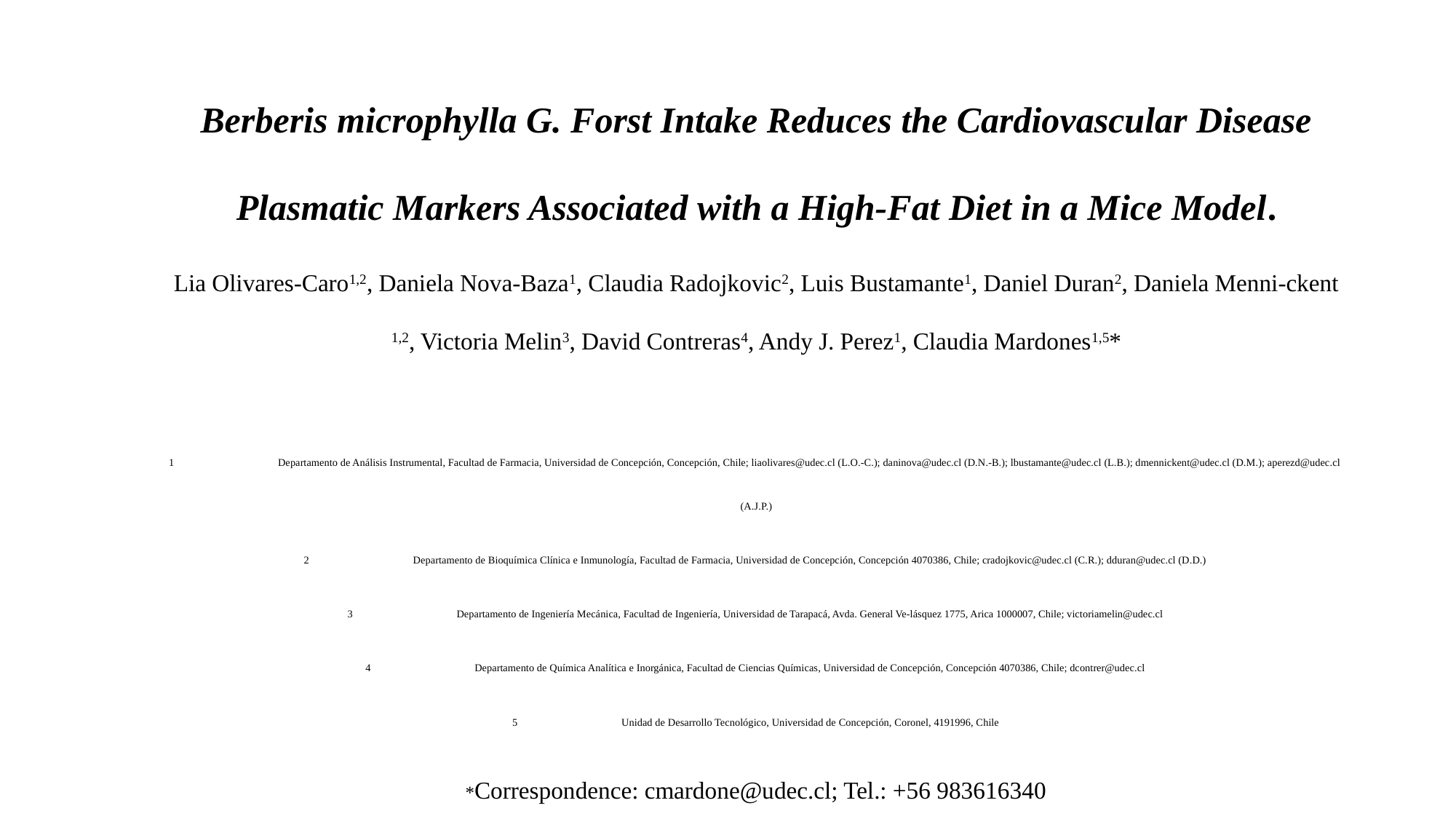

Berberis microphylla G. Forst Intake Reduces the Cardiovascular Disease Plasmatic Markers Associated with a High-Fat Diet in a Mice Model.
Lia Olivares-Caro1,2, Daniela Nova-Baza1, Claudia Radojkovic2, Luis Bustamante1, Daniel Duran2, Daniela Menni-ckent 1,2, Victoria Melin3, David Contreras4, Andy J. Perez1, Claudia Mardones1,5*
1	Departamento de Análisis Instrumental, Facultad de Farmacia, Universidad de Concepción, Concepción, Chile; liaolivares@udec.cl (L.O.-C.); daninova@udec.cl (D.N.-B.); lbustamante@udec.cl (L.B.); dmennickent@udec.cl (D.M.); aperezd@udec.cl (A.J.P.)
2	Departamento de Bioquímica Clínica e Inmunología, Facultad de Farmacia, Universidad de Concepción, Concepción 4070386, Chile; cradojkovic@udec.cl (C.R.); dduran@udec.cl (D.D.)
3	Departamento de Ingeniería Mecánica, Facultad de Ingeniería, Universidad de Tarapacá, Avda. General Ve-lásquez 1775, Arica 1000007, Chile; victoriamelin@udec.cl
4	Departamento de Química Analítica e Inorgánica, Facultad de Ciencias Químicas, Universidad de Concepción, Concepción 4070386, Chile; dcontrer@udec.cl
5	Unidad de Desarrollo Tecnológico, Universidad de Concepción, Coronel, 4191996, Chile
*Correspondence: cmardone@udec.cl; Tel.: +56 983616340

## Slide 2
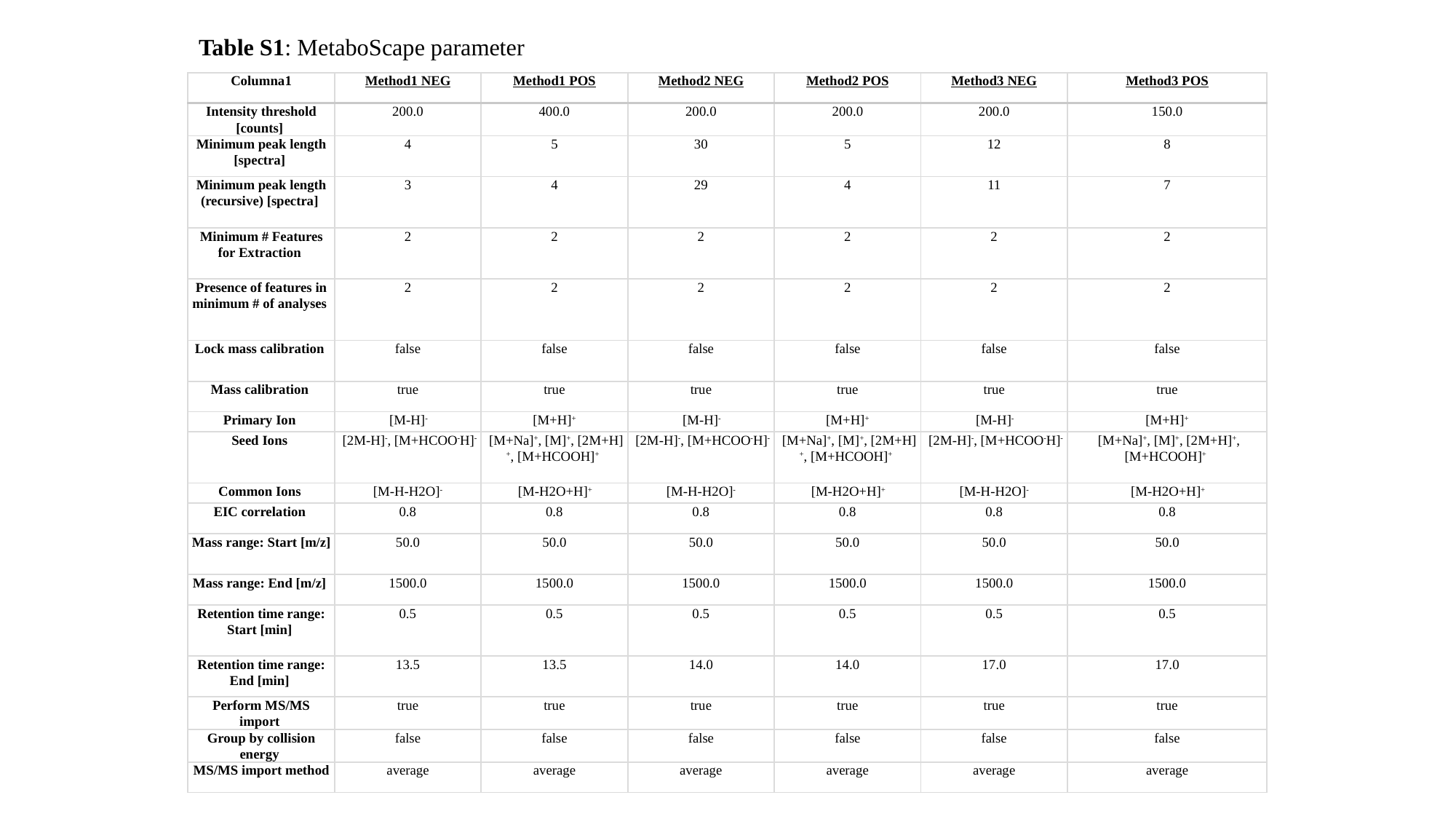

Table S1: MetaboScape parameter
| Columna1 | Method1 NEG | Method1 POS | Method2 NEG | Method2 POS | Method3 NEG | Method3 POS |
| --- | --- | --- | --- | --- | --- | --- |
| Intensity threshold [counts] | 200.0 | 400.0 | 200.0 | 200.0 | 200.0 | 150.0 |
| Minimum peak length [spectra] | 4 | 5 | 30 | 5 | 12 | 8 |
| Minimum peak length (recursive) [spectra] | 3 | 4 | 29 | 4 | 11 | 7 |
| Minimum # Features for Extraction | 2 | 2 | 2 | 2 | 2 | 2 |
| Presence of features in minimum # of analyses | 2 | 2 | 2 | 2 | 2 | 2 |
| Lock mass calibration | false | false | false | false | false | false |
| Mass calibration | true | true | true | true | true | true |
| Primary Ion | [M-H]- | [M+H]+ | [M-H]- | [M+H]+ | [M-H]- | [M+H]+ |
| Seed Ions | [2M-H]-, [M+HCOO-H]- | [M+Na]+, [M]+, [2M+H]+, [M+HCOOH]+ | [2M-H]-, [M+HCOO-H]- | [M+Na]+, [M]+, [2M+H]+, [M+HCOOH]+ | [2M-H]-, [M+HCOO-H]- | [M+Na]+, [M]+, [2M+H]+, [M+HCOOH]+ |
| Common Ions | [M-H-H2O]- | [M-H2O+H]+ | [M-H-H2O]- | [M-H2O+H]+ | [M-H-H2O]- | [M-H2O+H]+ |
| EIC correlation | 0.8 | 0.8 | 0.8 | 0.8 | 0.8 | 0.8 |
| Mass range: Start [m/z] | 50.0 | 50.0 | 50.0 | 50.0 | 50.0 | 50.0 |
| Mass range: End [m/z] | 1500.0 | 1500.0 | 1500.0 | 1500.0 | 1500.0 | 1500.0 |
| Retention time range: Start [min] | 0.5 | 0.5 | 0.5 | 0.5 | 0.5 | 0.5 |
| Retention time range: End [min] | 13.5 | 13.5 | 14.0 | 14.0 | 17.0 | 17.0 |
| Perform MS/MS import | true | true | true | true | true | true |
| Group by collision energy | false | false | false | false | false | false |
| MS/MS import method | average | average | average | average | average | average |

## Slide 3
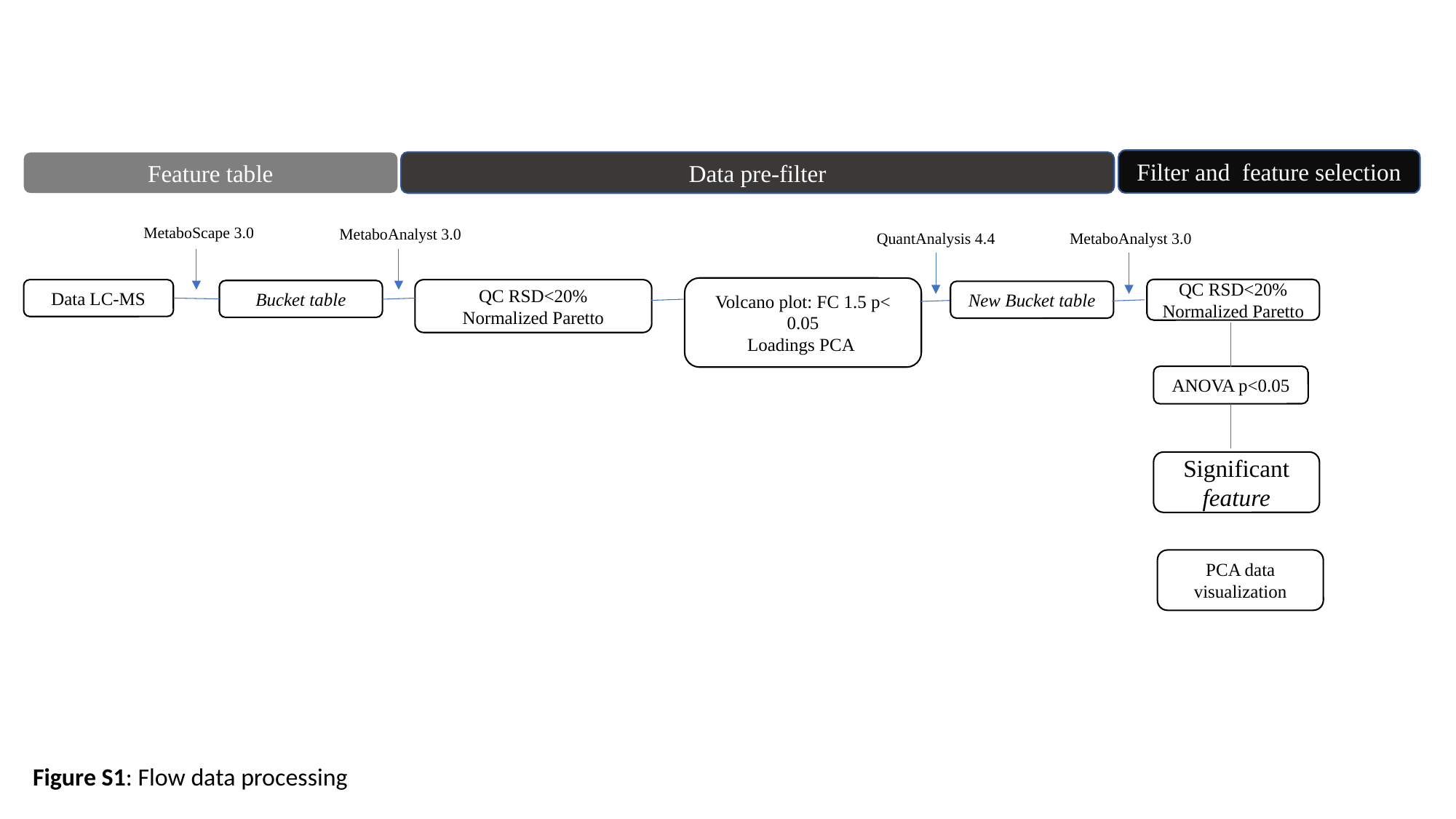

Filter and feature selection
Data pre-filter
Feature table
MetaboScape 3.0
MetaboAnalyst 3.0
QuantAnalysis 4.4
MetaboAnalyst 3.0
Volcano plot: FC 1.5 p< 0.05
Loadings PCA
QC RSD<20%
Normalized Paretto
Data LC-MS
QC RSD<20%
Normalized Paretto
Bucket table
New Bucket table
ANOVA p<0.05
Significant feature
PCA data visualization
Figure S1: Flow data processing

## Slide 4
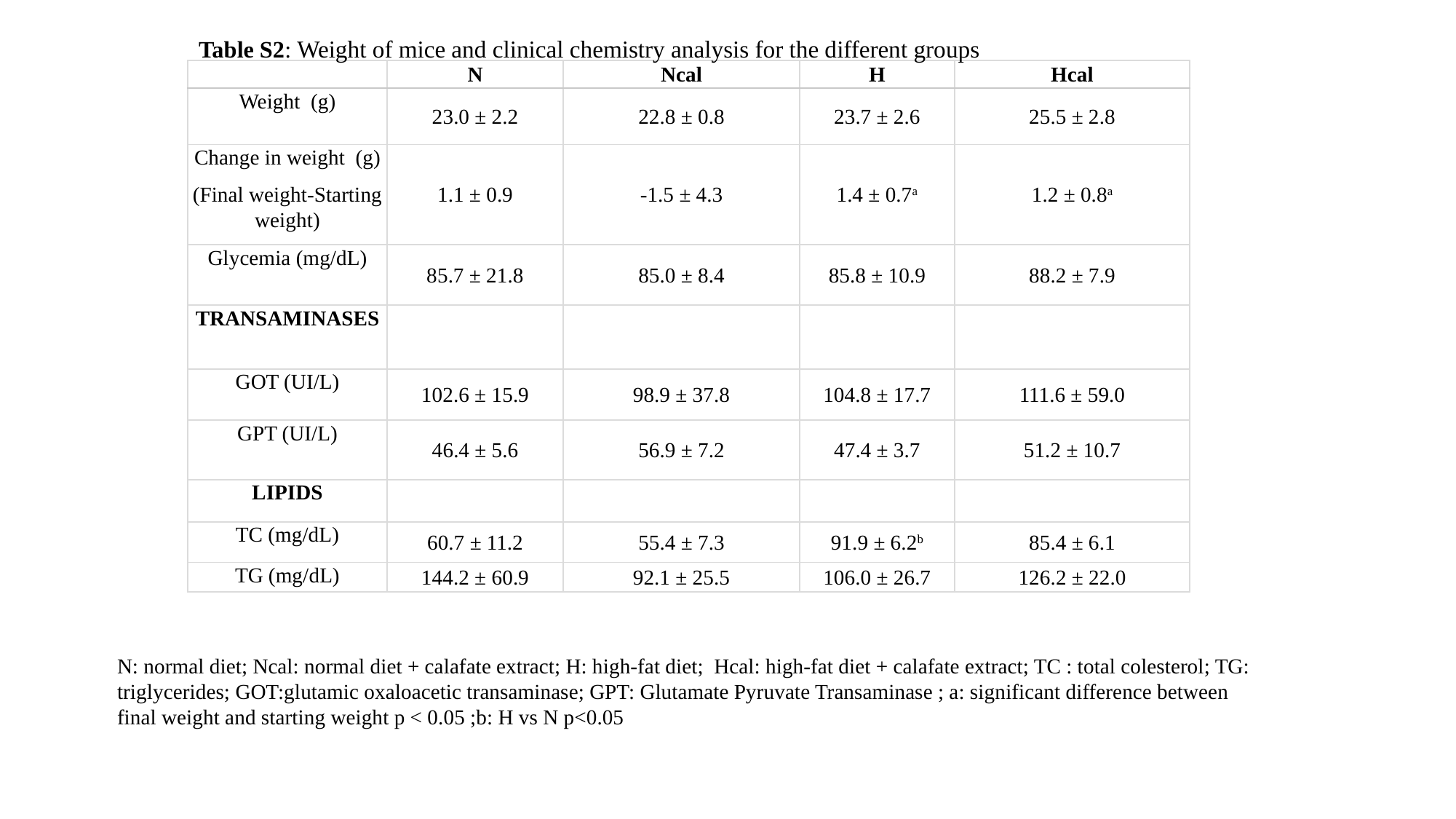

Table S2: Weight of mice and clinical chemistry analysis for the different groups
| | N | Ncal | H | Hcal |
| --- | --- | --- | --- | --- |
| Weight (g) | 23.0 ± 2.2 | 22.8 ± 0.8 | 23.7 ± 2.6 | 25.5 ± 2.8 |
| Change in weight (g) (Final weight-Starting weight) | 1.1 ± 0.9 | -1.5 ± 4.3 | 1.4 ± 0.7a | 1.2 ± 0.8a |
| Glycemia (mg/dL) | 85.7 ± 21.8 | 85.0 ± 8.4 | 85.8 ± 10.9 | 88.2 ± 7.9 |
| TRANSAMINASES | | | | |
| GOT (UI/L) | 102.6 ± 15.9 | 98.9 ± 37.8 | 104.8 ± 17.7 | 111.6 ± 59.0 |
| GPT (UI/L) | 46.4 ± 5.6 | 56.9 ± 7.2 | 47.4 ± 3.7 | 51.2 ± 10.7 |
| LIPIDS | | | | |
| TC (mg/dL) | 60.7 ± 11.2 | 55.4 ± 7.3 | 91.9 ± 6.2b | 85.4 ± 6.1 |
| TG (mg/dL) | 144.2 ± 60.9 | 92.1 ± 25.5 | 106.0 ± 26.7 | 126.2 ± 22.0 |
N: normal diet; Ncal: normal diet + calafate extract; H: high-fat diet; Hcal: high-fat diet + calafate extract; TC : total colesterol; TG: triglycerides; GOT:glutamic oxaloacetic transaminase; GPT: Glutamate Pyruvate Transaminase ; a: significant difference between final weight and starting weight p < 0.05 ;b: H vs N p<0.05

## Slide 5
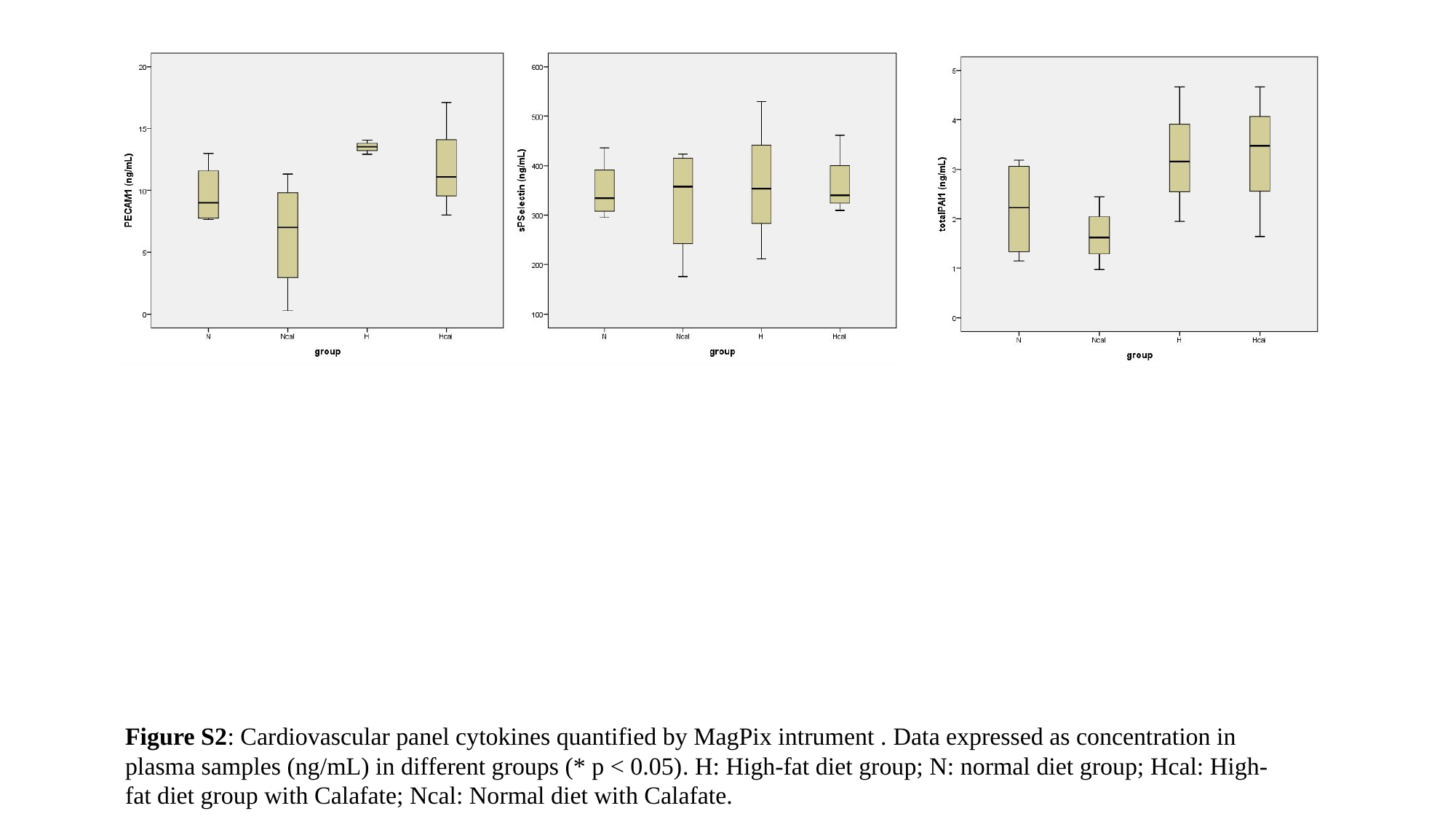

Figure S2: Cardiovascular panel cytokines quantified by MagPix intrument . Data expressed as concentration in plasma samples (ng/mL) in different groups (* p < 0.05). H: High-fat diet group; N: normal diet group; Hcal: High-fat diet group with Calafate; Ncal: Normal diet with Calafate.

## Slide 6
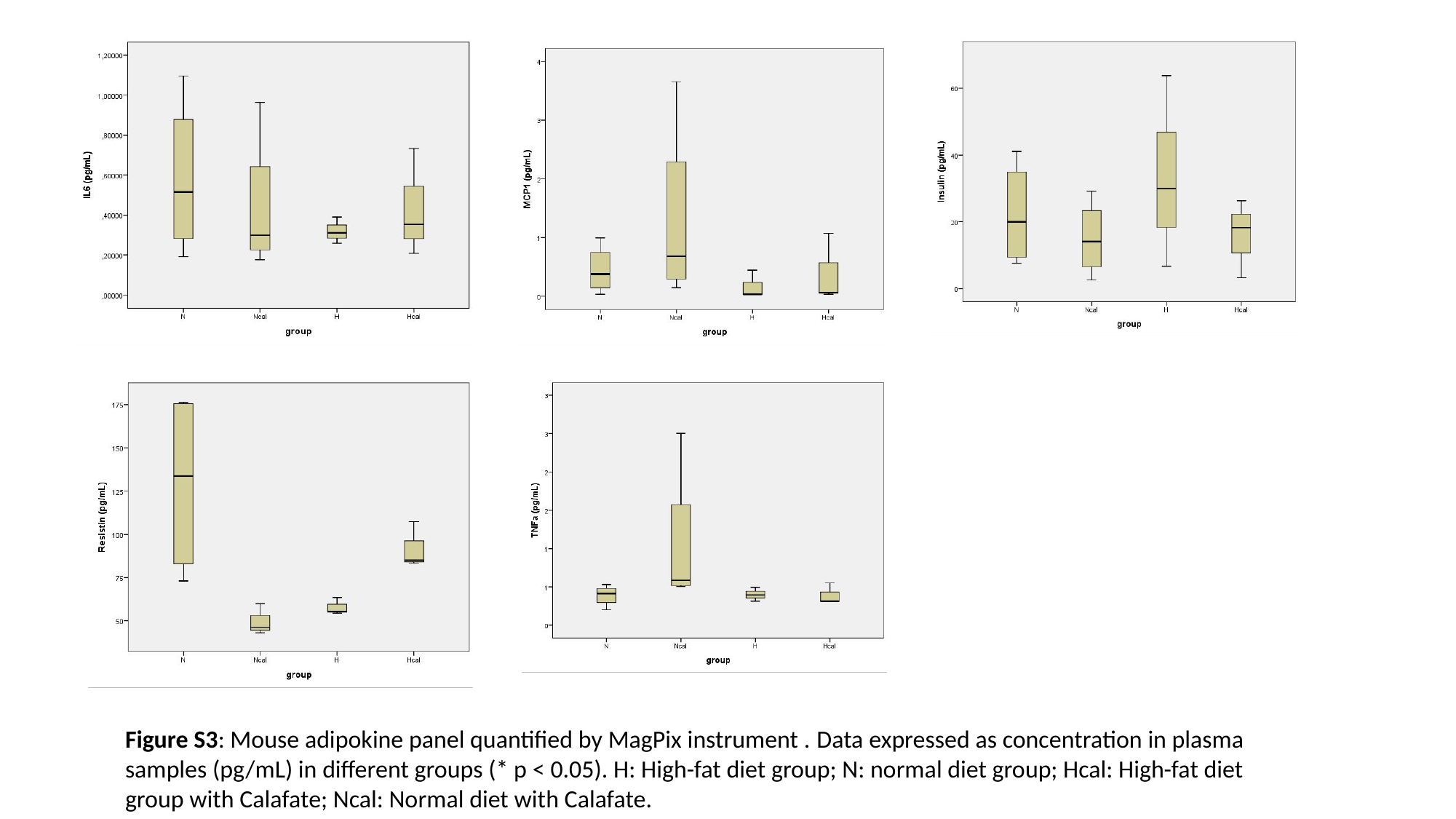

Figure S3: Mouse adipokine panel quantified by MagPix instrument . Data expressed as concentration in plasma samples (pg/mL) in different groups (* p < 0.05). H: High-fat diet group; N: normal diet group; Hcal: High-fat diet group with Calafate; Ncal: Normal diet with Calafate.

## Slide 7
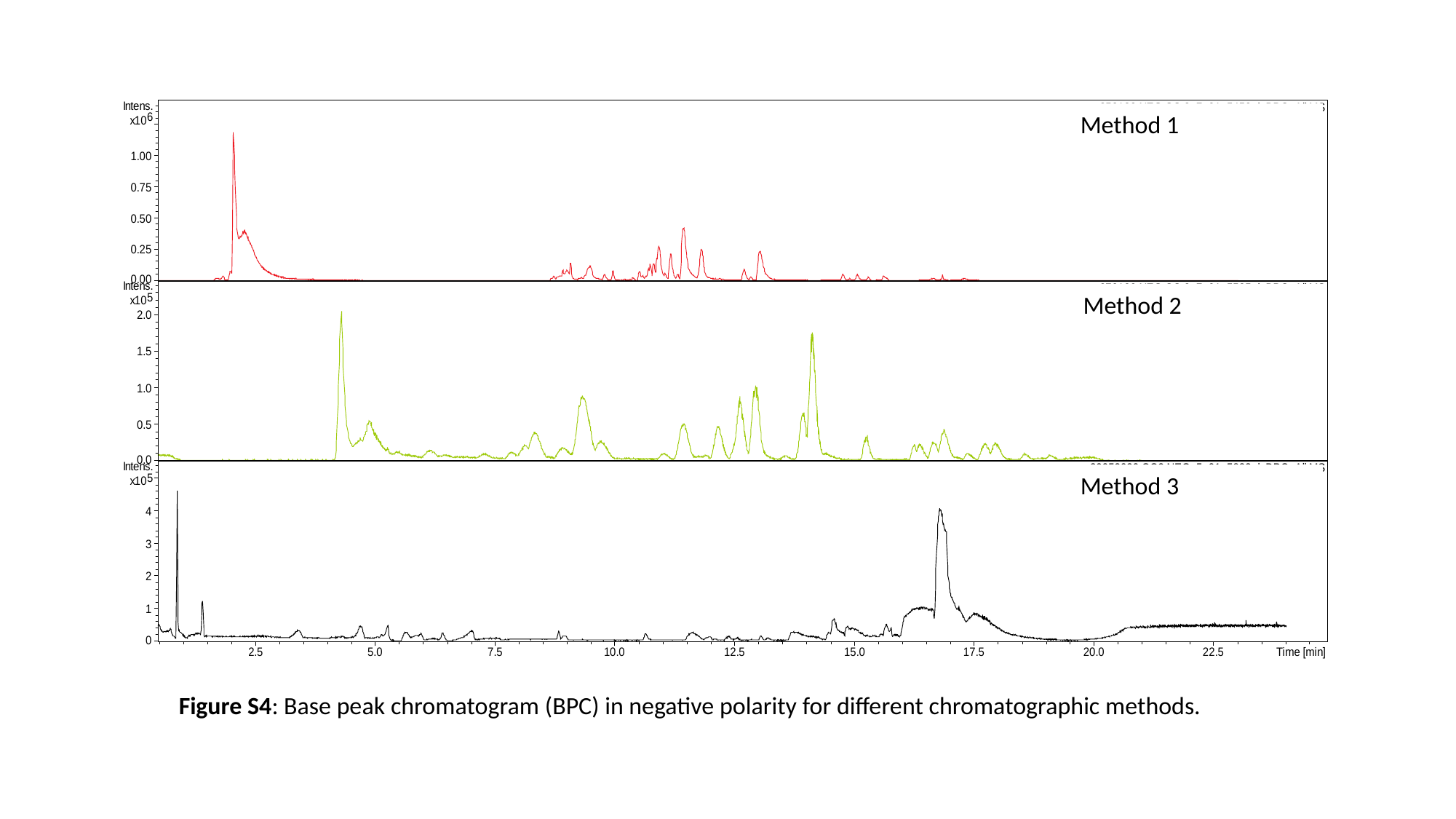

Method 1
Method 2
Method 3
Figure S4: Base peak chromatogram (BPC) in negative polarity for different chromatographic methods.

## Slide 8
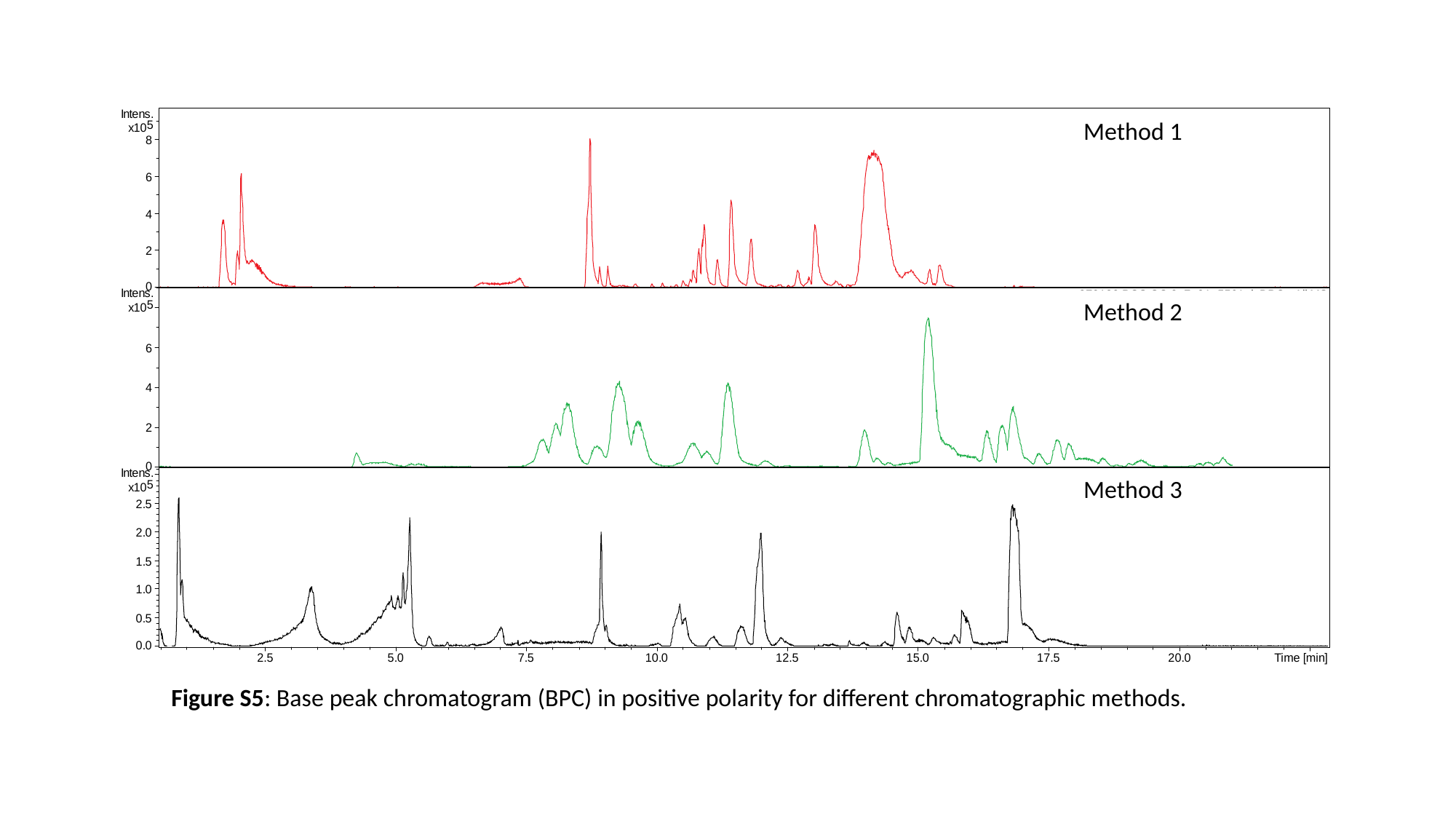

Method 1
Method 2
Method 3
Figure S5: Base peak chromatogram (BPC) in positive polarity for different chromatographic methods.

## Slide 9
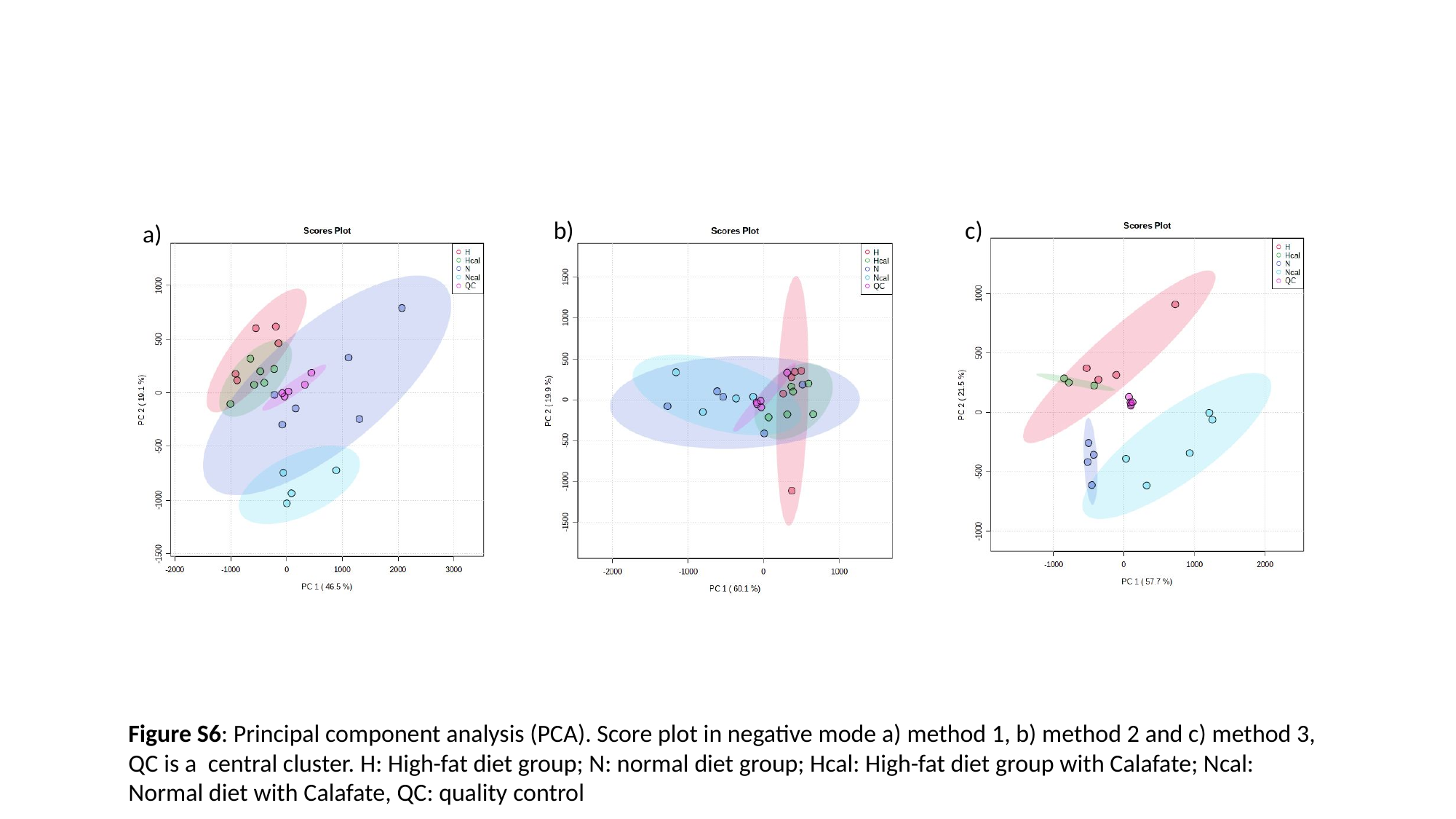

b)
c)
a)
Figure S6: Principal component analysis (PCA). Score plot in negative mode a) method 1, b) method 2 and c) method 3, QC is a central cluster. H: High-fat diet group; N: normal diet group; Hcal: High-fat diet group with Calafate; Ncal: Normal diet with Calafate, QC: quality control

## Slide 10
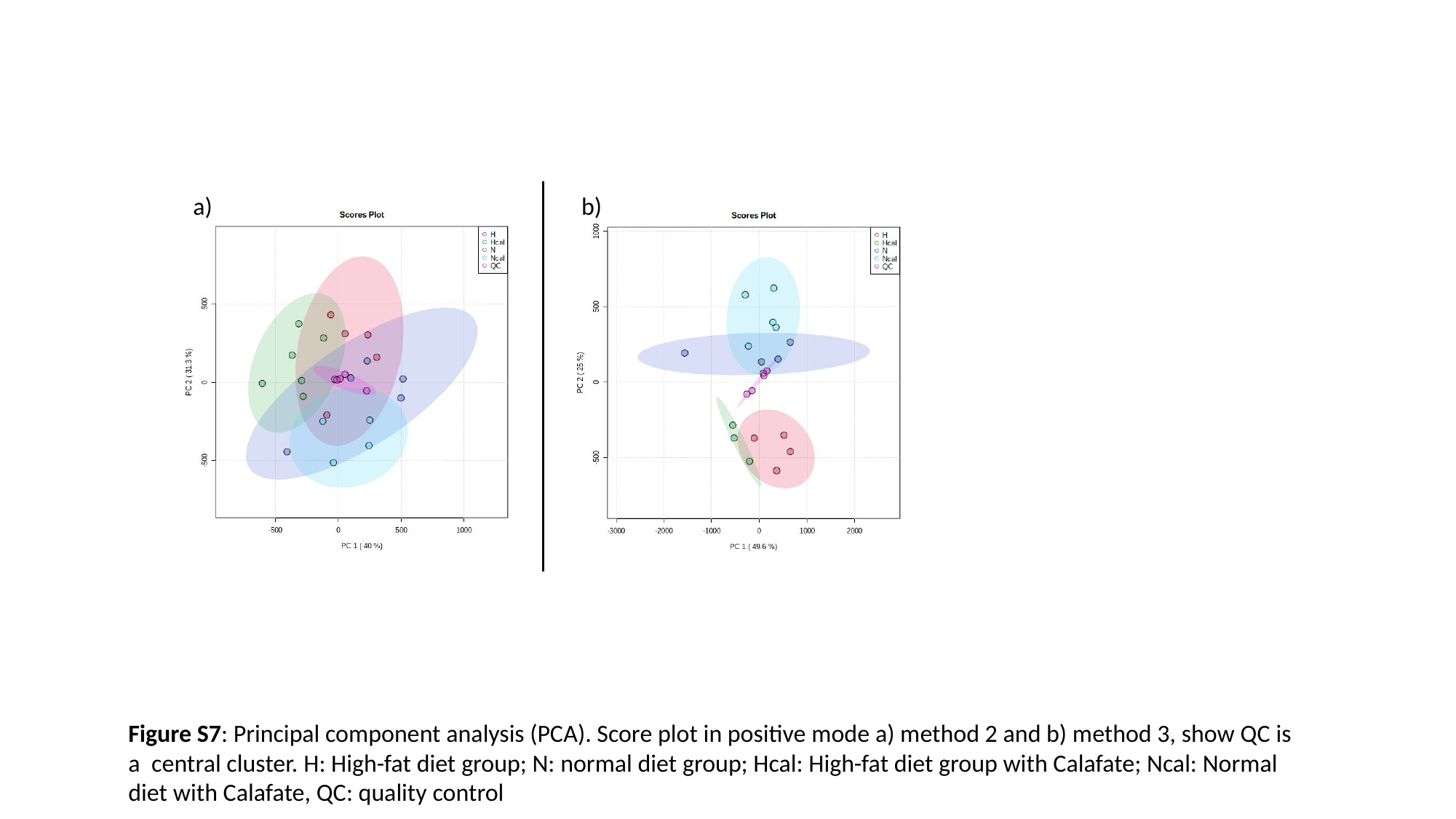

a)
b)
Figure S7: Principal component analysis (PCA). Score plot in positive mode a) method 2 and b) method 3, show QC is a central cluster. H: High-fat diet group; N: normal diet group; Hcal: High-fat diet group with Calafate; Ncal: Normal diet with Calafate, QC: quality control

## Slide 11
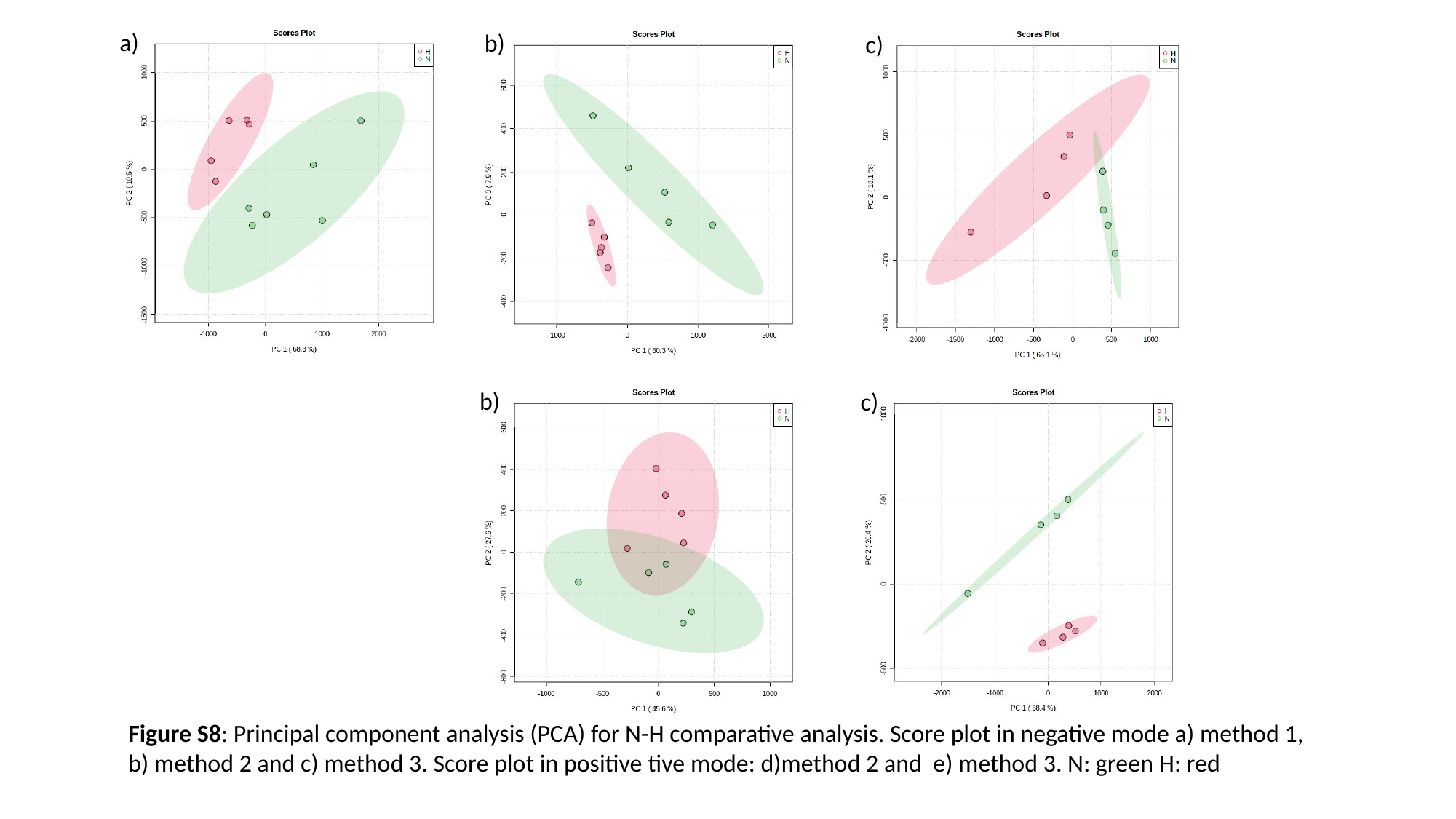

a)
b)
c)
b)
c)
Figure S8: Principal component analysis (PCA) for N-H comparative analysis. Score plot in negative mode a) method 1, b) method 2 and c) method 3. Score plot in positive tive mode: d)method 2 and e) method 3. N: green H: red

## Slide 12
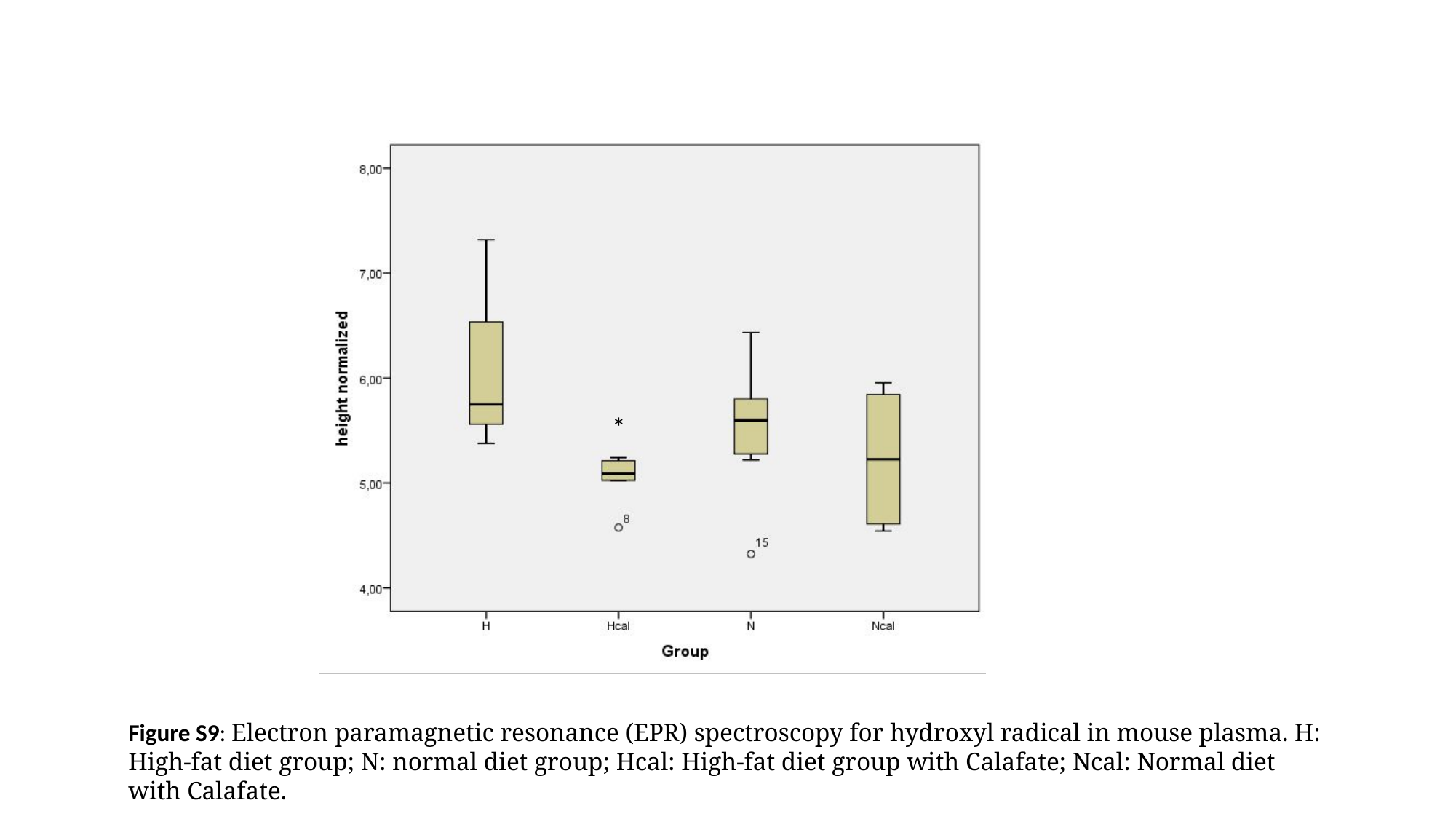

*
Figure S9: Electron paramagnetic resonance (EPR) spectroscopy for hydroxyl radical in mouse plasma. H: High-fat diet group; N: normal diet group; Hcal: High-fat diet group with Calafate; Ncal: Normal diet with Calafate.

## Slide 13
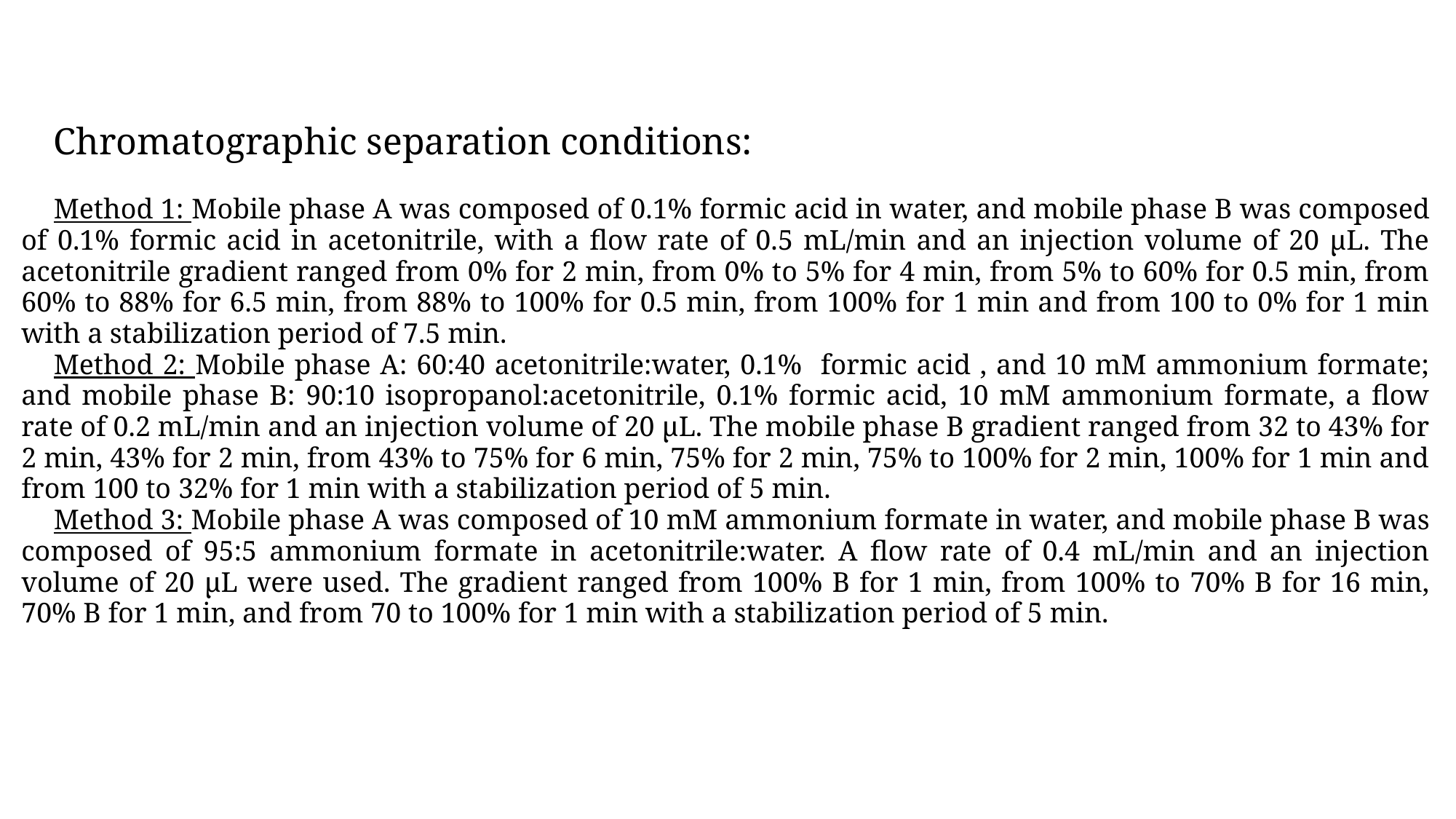

Chromatographic separation conditions:
Method 1: Mobile phase A was composed of 0.1% formic acid in water, and mobile phase B was composed of 0.1% formic acid in acetonitrile, with a flow rate of 0.5 mL/min and an injection volume of 20 μL. The acetonitrile gradient ranged from 0% for 2 min, from 0% to 5% for 4 min, from 5% to 60% for 0.5 min, from 60% to 88% for 6.5 min, from 88% to 100% for 0.5 min, from 100% for 1 min and from 100 to 0% for 1 min with a stabilization period of 7.5 min.
Method 2: Mobile phase A: 60:40 acetonitrile:water, 0.1% formic acid , and 10 mM ammonium formate; and mobile phase B: 90:10 isopropanol:acetonitrile, 0.1% formic acid, 10 mM ammonium formate, a flow rate of 0.2 mL/min and an injection volume of 20 μL. The mobile phase B gradient ranged from 32 to 43% for 2 min, 43% for 2 min, from 43% to 75% for 6 min, 75% for 2 min, 75% to 100% for 2 min, 100% for 1 min and from 100 to 32% for 1 min with a stabilization period of 5 min.
Method 3: Mobile phase A was composed of 10 mM ammonium formate in water, and mobile phase B was composed of 95:5 ammonium formate in acetonitrile:water. A flow rate of 0.4 mL/min and an injection volume of 20 μL were used. The gradient ranged from 100% B for 1 min, from 100% to 70% B for 16 min, 70% B for 1 min, and from 70 to 100% for 1 min with a stabilization period of 5 min.
